# Designing zero-dimensional dimer-type all-inorganic perovskites for ultra-fast switching memory

**DOI:** 10.1038/s41467-021-23871-w

**Published:** 2021-06-10

**Authors:** Youngjun Park, Seong Hun Kim, Donghwa Lee, Jang-Sik Lee

**Affiliations:** 1 Department of Materials Science and Engineering, Pohang University of Science and Technology (POSTECH), Pohang, 37673 Korea; 2 Division of Advanced Materials Science, Pohang University of Science and Technology (POSTECH), Pohang, Korea

**Keywords:** Electrical and electronic engineering, Information storage, Bionanoelectronics

## Abstract

Resistive switching memory that uses halide perovskites (HP) has been considered as next-generation storage devices due to low operation voltage and high on/off ratio. However, the memory still faces challenges for stable operation with fast switching speed, which hinders the practical application. Thus, it should be considered from the stage of designing the HP for memory applications. Here, we design the perovskite memory using a high-throughput screening based on first-principles calculations. Total 696 compositions in four different crystal structures are investigated and essential parameters including stability, vacancy formation, and migration are considered as the descriptor. We select dimer-Cs_3_Sb_2_I_9_ as an optimal HP for memory; the device that uses dimer-Cs_3_Sb_2_I_9_ has ultra-fast switching speed (~20 ns) compared to the device that uses layer-Cs_3_Sb_2_I_9_ (>100 ns). The use of lead-free perovskite avoids environmental problems caused by lead in perovskite. These results demonstrate the feasibility to design the memory with ultra-fast switching speed.

## Abstract (web summary)

Halide perovskite has been applied for resistive switching memory devices, but there are challenges remained to achieve practical application. By using high-throughput screening based on first-principles calculations, the authors discover that lead-free dimer-Cs_3_Sb_2_I_9_ meets the requirements, which exhibits switching speed of 20 ns.

## Introduction

The development of halide perovskites (HPs) has been witnessed in various research areas including electronic and optoelectronic devices because of their excellent properties such as high carrier mobility, high absorption coefficient, and adjustable bandgaps^1–7^. HPs have current–voltage (*I*–*V*) hysteresis by migration of ions or defects, which can induce resistive switching behaviors^8–10^. The implementation of the resistive switching behaviors enables various applications such as memory and neuromorphic devices^11–14^. Among various applications, HPs have been evaluated for use in resistive switching memory (RSM) because of high on/off ratio with multilevel data storage, low operation voltage, and forming-free properties^15–19^. These advantages of RSM that uses HP permit implementation as artificial synapses for neuromorphic application^11,18^. However, the RSM still faces challenges for stable operation with fast switching speed. The HP RSM devices can be operated with a limited switching speed, whereas RSM devices that use oxide materials can be operated by using a pulse of sub-nanosecond^20–22^. The switching speed of HP-based RSM is limited even in a small memory cell compared to that of oxide-based memory^23,24^. This limited speed hinders the practical application of HP to RSM despite the advantages of HP as a memory. HP RSM can be operated by formation and rupture of conductive filaments by migration of defects (halide-ion vacancies)^25^. Therefore, to improve the switching speed, the migration of defects in the HP should be understood, and HP in which defects can easily move should be identified. This process should be considered from the stage of designing the HP for RSM.

The use of a computational approach to find an optimal HP for RSM can screen and reduce the number of possible candidate materials, and thereby avoid the human effort, time, and cost of fabricating and testing all possible combinations. The first-principles-based approaches have been spotlighted as a powerful tool to accelerate materials researches. In various fields, high-throughput screening methods that use density functional theory (DFT) have been successfully applied^26,27^, but it has not been used to evaluate materials for use in RSM. In addition, the HP RSM devices have been widely developed in three-dimensional (3D) and two-dimensional (2D) layered structures containing lead (Pb)^28,29^, which is environmentally toxic and this component hinders commercialization of devices that use HP. Also, Pb can induce instability of HPs under air and humidity conditions^30,31^. Tin (Sn) and germanium (Ge) have been evaluated as less-toxic replacements for Pb^32,33^, but the HPs have low chemical stability because Sn^2+^ and Ge^2+^ oxidize easily to Sn^4+^ and Ge^4+^ in ambient air and humidity^32,34^. Other Pb-free all-inorganic materials have been evaluated as memory devices^35–37^, but an appropriate Pb-free HP for stable and high-speed RSM is still required.

In this work, to design a Pb-free HP for RSM with fast switching speed, we combine high-throughput screening and experimental verification. We apply high-throughput screening using first-principles DFT calculations to select promising HPs for RSM. We identify seven candidate HPs considering formation energy for high stability and defect-formation energy for filament formation. Then, of these seven compositions, we calculate the vacancy-migration energy to identify three candidates that are expected to have a fast switching speed, then finally select Cs_3_Sb_2_I_9_ with a dimer structure. Experimentally, Cs_3_Sb_2_I_9_ can be synthesized in two forms: a dimer structure and a layered structure^38,39^. Our calculation results show that the two structures have similar formation energies and defect-formation energies, but dimer-Cs_3_Sb_2_I_9_ is expected to have faster switching speed because the vacancy-migration barrier is lower than that of layer-Cs_3_Sb_2_I_9_. To verify our calculation results, we fabricate the RSM device using the dimer-Cs_3_Sb_2_I_9_ and compare its resistive switching behavior to RSM using the layer-Cs_3_Sb_2_I_9_. Fabricated RSM that uses dimer-Cs_3_Sb_2_I_9_ shows a fast switching speed of ~20 ns, whereas the RSM that uses layer-Cs_3_Sb_2_I_9_ shows a limited switching speed (>100 ns). These results are consistent with the vacancy-migration energy obtained from the DFT calculations. These studies confirm that the combined method of high-throughput screening and experimental verification can be a promising route to design materials for HP RSM devices.

## Results

### Material design by high-throughput screening

**Fig. 1:**
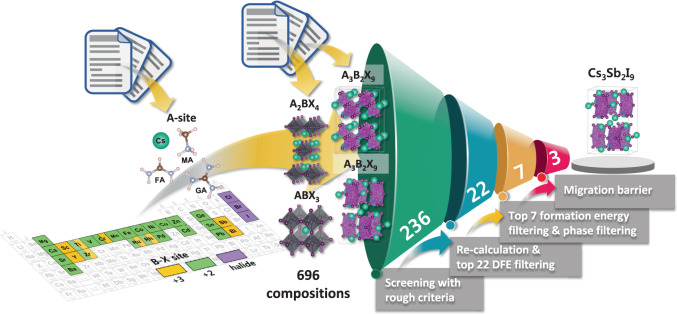
Four-step screening to identify HP materials for RSM. Four structures that are considered in the four-step high-throughout screening calculation. For trivalent B-site cation, dimer-type A_3_B_2_X_9_ structure and layer-type A_3_B_2_X_9_ structure are used. For divalent B-site cation, layered A_2_BX_4_ structure and cubic ABX_3_ structure are used. Bold numbers in truncated cones indicate the number of compounds that meet the screening criteria.

To find optimal materials for use in Pb-free HP for RSM with fast switching speed, we chose four backbone structures: dimer-type A_3_B_2_X_9_, layer-type A_3_B_2_X_9_, layer-type A_2_BX_4_, and cubic ABX_3_ (Fig. 1). For dimer-type A_3_B_2_X_9_ crystal systems, the crystal structure of Cs_3_Bi_2_I_9_ with P6_3_/mmc space group was used as the backbone structure^40^; it has bioctahedral dimers composed of B-X ions. For layer-type A_3_B_2_X_9_ crystal systems, Cs_3_Sb_2_Br_9_ crystal structure ($P\bar{3}m1$) was considered for the backbone structure; it has corner-connected bilayer octahedrons^41^. For A_2_BX_4_ crystal systems, the Ruddlesden–Popper HP structure was considered; it has corner-connected single-layer octahedra composed of B-X ions. Between two kinds of A_2_BX_4_ arrangements (staggered, eclipsed), the staggered arrangement was chosen for the backbone structure because small molecules were prefered to be staggered arrangement^42^. For ABX_3_ structure, we used CsPbI_3_ crystal structure (${Pm}\bar{3}m$), which has been used as a material for RSM^43^. First-principles-based high-throughput screening was performed by changing the composition of four crystal systems. At the A-site, we used four monovalent cations [CH_3_NH_3_^+^ (MA^+^), HC(NH_2_)_2_^+^ (FA^+^), C(NH_2_)_3_^+^ (GA^+^), and Cs^+^), which are commonly used in HP. At the B-site cations, 20 divalent cations (Mg^2+^, Ca^2+^, Sr^2+^, Ba^2+^, Ti^2+^, Zr^2+^, Cr^2+^, V^2+^, Fe^2+^, Mn^2+^, Co^2+^, Cu^2+^, Ni^2+^, Zn^2+^, Rh^2+^, Pd^2+^, Cd^2+^, Fe^2+^, Sn^2+^, and Pb^2+^) and 9 trivalent cations (Sc^3+^, Ti^3+^, Cr^3+^, Y^3+^, Zr^3+^, Ru^3+^, Rh^3+^, Sb^3+^, and Bi^3+^) were chosen. According to the stoichiometry of the crystal systems, trivalent or divalent cations were used in the B-site. At the X-site, we used three halide anions (Cl^–^, Br^–^, and I^–^). As a result, we considered a total of 696 combinations (240 compounds for each layer-A_2_BX_4_ and ABX_3_, and 108 compounds for each dimer-A_3_B_2_X_9_ and layer-A_3_B_2_X_9_) in this study. To design an optimal HP to apply RSM, we selected formation energy, halide vacancy defect-formation energy, and vacancy-migration barrier as descriptors for high-throughput screening. First, formation energy (*E*_form_) (eV f.u.^−1^, where f.u. is formula unit) was considered because it could represent material stability^44,45^. *E*_form_ of each compound can be calculated as:

1 $${E}_{{\rm{form}}}={E}_{{\rm{bulk}}}^{{\rm{tot}}}-\left(a{E}_{{\rm{AX}}}^{{\rm{tot}}}+b{E}_{{{\rm{BX}}}_{n}}^{{\rm{tot}}}\right)$$

where ${E}_{{\rm{bulk}}}^{{\rm{tot}}}$ is the total energy of the A-B-X ternary compound. ${E}_{{\rm{AX}}}^{{\rm{tot}}}$ is the total energy of precursor AX, and ${E}_{{{\rm{BX}}}_{n}}^{{\rm{tot}}}$ is the total energy of precursor BX_*n*_. The widely used binary compounds in the synthesis of HP (AX and BX_*n*_) were chosen as the precursors^46,47^. The subscript *n* in BX_*n*_ was determined by the charge state of B ion; if it is trivalent, *n* = 3 and if it is divalent, *n* = 2. The stoichiometry of the A-B-X compound was maintained by appropriately determining *a* and *b*: for A_3_B_2_X_9_, *a* = 3, *b* = 2; ABX_3_, *a* = 1, *b* = 1; and for A_2_BX_4_, *a* = 2, *b* = 1. Second, we used halide vacancy defect-formation energy (DFE [*V*_*X*_], eV) because previous studies showed that it was most correlated with the operation of RSM device^48^. DFE [*V*_*X*_] can be calculated as

2 $${\rm{DFE}}\left[{V}_{X}\right]={E}_{{\rm{tot}}}\left[{V}_{X}\right]-{E}_{{\rm{bulk}}}^{{\rm{tot}}}+{\mu }_{x}$$

where *E*_tot_ [*V*_*X*_] is the total energy of the system with single defect (*V*_*X*_), ${E}_{{\rm{bulk}}}^{{\rm{tot}}}$ is the total energy of a perfect A-B-X ternary system, and *μ*_*x*_ is the chemical potential of X anion. Third, we considered the migration barrier of *V*_*X*_ (*E*_mig_ [*V*_*X*_]) as a descriptor for the switching speed of RSM device, because the migration barrier of halide vacancies could affect the switching behavior of RSM device^25,49–51^. We note here that the migration barrier of *V*_*X*_ is not the only factor that determines the switching speed of RSM device. Other factors can also affect the switching speed of RSM device. Examples include process-dependent materials characteristics such as defect concentration, morphology, grain size, homogeneity, and device structure^52^. However, the migration barrier of a halide vacancy is one of the main parameters that can affect the operation speed of RSM devices^53,54^, so it can be used to screen candidate materials. In the crystal structures considered in this study, a halide vacancy can follow either an intra-octahedron path or an inter-octahedron path (Supplementary Fig. 1). Additional calculations were performed to identify the rate-limiting migration pathway of *V*_*X*_. DFT results predicted that inter-octahedron *E*_mig_ [*V*_*X*_] was higher than intra-octahedron *E*_mig_ [*V*_*X*_] (Supplementary Table 1). Thus, we selected the inter-octahedron *E*_mig_ [*V*_*X*_] as the last descriptor.

**Fig. 2:**
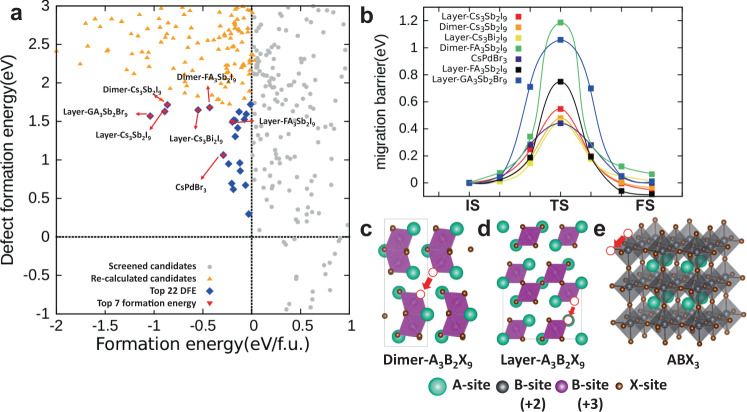
High-throughput screening for HP RSM. **a** Formation energies and halide vacancy defect-formation energy of 696 types of candidates. Through four-step screening, the candidates are filtered by 236 possible compositions (yellow), top 22 by defect-formation energy filtering (blue), and top 7 by formation energy and phase filtering (red). The gray circles are excluded candidates due to their positive formation energy. **b** A vacancy-migration barrier of top seven candidates (IS initial state, TS transition state, FS final state). Schematic structures of **c** dimer-A_3_B_2_X_9_, **d** layer-A_3_B_2_X_9_, and (**e**) ABX_3_ (red circle: halide vacancy site, red arrows: vacancy-migration paths). The rectangular boxes in schematic structures are the unit cell of crystal structure.

The high-throughput screening was conducted in four steps. During each screening step, the number of candidate compounds was reduced, and finally we selected one that was expected to be the best for use in RSM. In the first step, to quickly exclude compounds that are unsuitable for RSM, we calculated *E*_form_ and DFE [*V*_*X*_] of 696 compounds with high force criteria (<0.05 eV/A) for geometry optimization (Fig. 2a, gray circles). Compounds with positive *E*_form_ were excluded because they were expected to be dissociated into precursors spontaneously. As a result, we reduced the number of candidate compounds to 236 species (Fig. 2a, orange triangles). In the second step, we recalculated the selected 236 candidates with low force criteria (<0.01 eV/A) then ranked them from lowest to highest according to DFE [*V*_*X*_]. Since the lower DFE [*V*_*X*_] leads to the higher *V*_*X*_ concentration, it can lower the operating voltage of the RSM device^55^. Thus, we chose the 22 compounds that had the lowest DFE [*V*_*X*_] among the 236 compounds. (Fig. 2a, blue diamonds). In the third step, we focused on the thermodynamic stability of candidate compounds. We ranked the recalculation results of 22 compounds from lowest to highest according to *E*_form_ to find compounds with high thermodynamic stability. Then, the 10 compounds with low *E*_form_ were selected among 22 compounds. Our screening that used DFT was performed for a specific crystal backbone structure, so we further checked whether the chosen compositions had different stable phases with other crystal structures. Among the ten selected compounds, three compounds (Cs_2_CuCl_4_, CsCuCl_3_, and Cs_2_PdBr_4_) that reported to exist in different crystal structures were also excluded^56–59^. Thus, seven possible compounds were selected for RSM in the third step (Fig. 2a, red down triangles). In the fourth step, we calculated *E*_mig_[*V*_*X*_] of the remaining compounds to find compounds that had a fast switching speed. Our calculation results predicted that three compounds [dimer-Cs_3_Sb_2_I_9_ (0.47 eV), layer-Cs_3_Bi_2_I_9_ (0.45 eV), CsPdBr_3_ (0.44 eV)] had lower *E*_mig_[*V*_*X*_] (<0.5 eV) than the others (Fig. 2b–e). Among the three selected compounds, the most probable candidate was selected by combining the results of all three descriptors. The three selected compounds showed similar vacancy-migration barriers, so we also compared *E*_form_ and DFE [*V*_*X*_], which could also affect the stability and device performance. Although CsPdBr_3_ and layer-Cs_3_Bi_2_I_9_ showed the low *E*_mig_ to migration of halide ion (0.44 and 0.47 eV, respectively), their *E*_form_ were higher than the others (CsPdBr_3_, −0.28 eV/f.u.; and layer-Cs_3_Bi_2_I_9_, −0.54 eV/f.u.). Therefore, CsPdBr_3_ and layer-Cs_3_Bi_2_I_9_ may not be able to form a high-quality and reliable RSM device. However, the *E*_form_ was much lower in dimer-Cs_3_Sb_2_I_9_ (−0.86 eV/f.u.) than in layer-Cs_3_Bi_2_I_9_ (−0.43 eV/f.u.). The low *E*_form_ may ensure the formation and reliability of the dimer-Cs_3_Sb_2_I_9_ RSM device, so we chose dimer-Cs_3_Sb_2_I_9_ as the most probable candidate for RSM device.

### Experimental verification

**Fig. 3:**
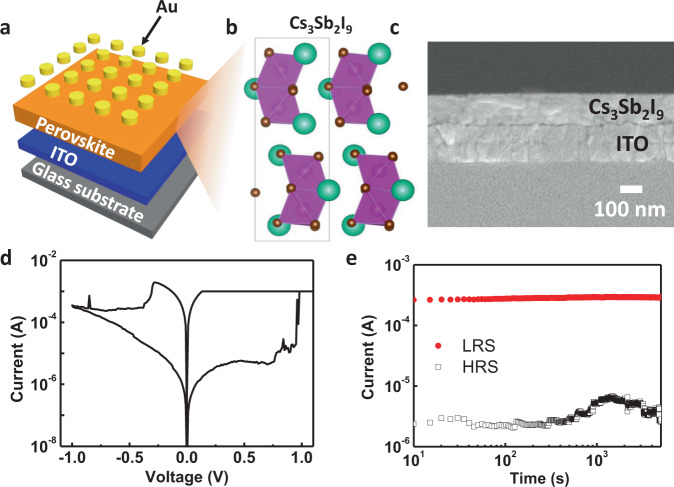
Schematic illustration and electrical characterstics of HP RSM. **a** Schematic illustration of RSM device using dimer-Cs_3_Sb_2_I_9_ deposited by a spin-coating process. **b** Schematic structure of dimer-Cs_3_Sb_2_I_9_ (atom colors: Cs = blue-green; Sb = purple; I = brown). The rectangular box in schematic structure is the unit cell of crystal structure. **c** Cross-sectional scanning-electron microscopy (SEM) image of dimer-Cs_3_Sb_2_I_9_ layer on ITO. **d** *I*–*V* characteristics of dimer-Cs_3_Sb_2_I_9_ RSM. **e** Data retention properties of the dimer-Cs_3_Sb_2_I_9_ RSM.

To verify the results of the calculations, we synthesized Cs_3_Sb_2_I_9_ with a dimer structure and used it in RSM device. The RSM device consisted of top Au electrode, bottom indium tin oxide (ITO) electrode, and a sandwiched thin-film layer of Cs_3_Sb_2_I_9_ (Fig. 3a). The dimer-Cs_3_Sb_2_I_9_ was deposited by spin-coating process using the antisolvent dripping method^39^. The Cs_3_Sb_2_I_9_ was composed of bioctahedral face-sharing [Sb_2_I_9_]^3−^ clusters (Fig. 3b). The deposited layer had a uniform thickness of 170 nm (Fig. 3c) with a dense and closely packed grain (Supplementary Fig. 2a). Atomic force microscopy (AFM) with a scan size of 5 × 5 μm showed that the Cs_3_Sb_2_I_9_ had a root mean square (RMS) roughness of 5.32 nm (Supplementary Fig. 2b, c). Also, we confirmed the grain size of dimer-Cs_3_Sb_2_I_9_ film. The average grain size of dimer-Cs_3_Sb_2_I_9_ film was 34 ± 4 nm, as calculated using the ASTM intercept procedure^60^. The crystalline structure of deposited Cs_3_Sb_2_I_9_ was investigated by X-ray diffraction (XRD) (Supplementary Fig. 3). The diffraction peaks were matched well with the dimer structure of Cs_3_Sb_2_I_9_ described prviously^38,39^. In X-ray photoelectron spectroscopy (XPS) core-level spectra, the Cs 3*d* spectrum exhibited paired peaks at 738.3 and 724.3 eV, the Sb 3*d* spectrum had peaks at 539.1 and 529.8 eV, and I 3*d* showed two peaks at 630.3 and 618.8 eV (Supplementary Fig. 4). These peaks are similar to previous reports for Cs_3_Sb_2_I_9_ films^61^.

The electrical characteristics of Au/dimer-Cs_3_Sb_2_I_9_/ITO were investigated to confirm the applicability of dimer-Cs_3_Sb_2_I_9_ to RSM device. During the measurement, the electrical signal was applied to the Au, and ITO was grounded. When voltage bias was swept in a positive direction, the current level was changed at 1 V, which suggested that the RSM was changed from a high-resistance state (HRS) to a low-resistance state (LRS) (Fig. 3d). When the voltage bias was swept in the negative direction, the LRS was returned to the initial HRS. We plotted cumulative probability distributions in the two resistance states to confirm the reliability of RSM device. The current levels of two states were measured at the read voltage (0.1 V) during 50 consecutive voltage sweeps (0 → 1 → 0 → −1 → 0 V). The device showed distinguishable HRS and LRS under repeated device operation (Supplementary Fig. 5). We measured data retention properties of LRS and HRS at a read voltage of 0.1 V. Both LRS and HRS remained stable for 5000 s without degradation (Fig. 3e). We measured the endurance characteristics using AC biases with positive triangular pulses that had a peak voltage of 2 V for the set process, and negative triangular pulses that had a peak of −1.6 V for the reset process. The width of each pulse was fixed at 10 μs and the current levels were measured under a read voltage of 0.1 V. The current level at a read voltage increased after the set process, but decreased after the reset process (Supplementary Fig. 6a, b). The device operated stably for 500 cycles (Supplementary Fig. 6c). We also measured device-to-device variation to quantify the reliability of the RSM device. The resistance states of 20 Cs_3_Sb_2_I_9_ RSM devices were measured at a read voltage. All devices had distinct HRS and LRS (Supplementary Fig. 7). These results indicated that the RSM could operate reliably. The stability of the device based on HPs in ambient air is a problem to be solved and it can be improved by using passivation layers^37,62^.

**Fig. 4:**
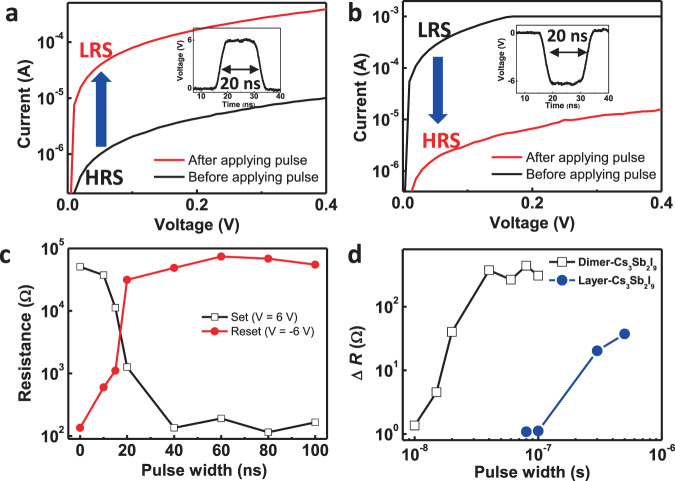
Switching speed of HP RSM. *I*–*V* characteristics of dimer-Cs_3_Sb_2_I_9_ RSM before and after application of (**a**) set pulse (6 V, 20 ns) and (**b**) reset pulse (−6 V, 20 ns). **c** Resistance changes of dimer-Cs_3_Sb_2_I_9_ RSM device with different pulse widths. Amplitudes of applied voltage pulses are fixed for set (6 V) and reset (−6 V) processes. **d** Resistance changes of dimer-Cs_3_Sb_2_I_9_ RSM and layer-Cs_3_Sb_2_I_9_ RSM devices by application of set pulses (6 V) with different pulse widths. Δ*R* is defined as the ratio between initial resistance and changed resistance after pulse application.

Fast operation is essential for the practical application of RSM devices, so HP RSM devices with fast switching speed should be achieved. To confirm the possibility of fast operation, we measured the switching speed of RSM device using dimer-Cs_3_Sb_2_I_9_. A voltage pulse with a pulse width of about 20 ns was applied to the device in parallel with the load resistor (100 Ω) using the pulse generator (Supplementary Fig. 8a). The series resistor of 500 Ω was additionally used to prevent the permanent breakdown of the device for the set process. Before the measurement, we confirmed that the voltage pulse of about 20 ns was properly applied to the device using the oscilloscope (Supplementary Fig. 8b, c). The switching speed of RSM was obtained by the required pulse width to change the resistance state. The voltage bias was swept from 0 to 0.4 V before and after pulse application to confirm the change of resistance state depending on the applied pulse. The voltage sweep range was lower than the operation voltage of RSM, and therefore could not affect the change of resistance. As the positive voltage pulse (6 V, 20 ns) was applied to the RSM device, the resistance state was changed from initial HRS to LRS (Fig. 4a). Also, the LRS was recovered to the initial HRS by application of the negative voltage pulse (−6 V, 20 ns) (Fig. 4b). The resistance change depended on the applied pulse width (Supplementary Fig. 9). The resistance change was observed under the pulse with 20-ns width and the resistance was completely changed when pulse with 40-ns width was applied. The resistance changes were plotted depending on the width of the applied pulse for set (6 V) and reset (−6 V) processes (Fig. 4c). The current level was also affected by the amplitude of voltage pulse. We applied the positive voltage pulses with different amplitudes (2, 4, 6, and 8 V) with a fixed pulse width of 20 ns (Supplementary Fig. 10). The change in current level increased as the amplitude of voltage pulse increased. To confirm the reliability of the device, we measured the data retention properties at LRS and HRS. The states were maintained without degradation for 10^3^ s (Supplementary Fig. 11). Also, we measured the endurance characteristics using nanosecond pulses (Supplementary Fig. 12). Set (6 V, 20 ns) and reset pulses (−6 V, 20 ns) were applied and the current level was measured using the read voltage pulse (20 μs, 0.1 V). The device was operated stably for 50 cycles. These results suggested that the dimer-Cs_3_Sb_2_I_9_ RSM device could be operated with a fast switching speed of 20 ns. For comparison, we also measured the switching speed of the RSM device that used layer-Cs_3_Sb_2_I_9_. DFT calculations predict that this device will have slower switching speed than the RSM device that uses dimer-Cs_3_Sb_2_I_9_, because layer-Cs_3_Sb_2_I_9_ has higher *E*_mig_ [*V*_*X*_] (0.57 eV) than dimer-Cs_3_Sb_2_I_9_ (0.47 eV). To verify this prediction, we deposited the layer-Cs_3_Sb_2_I_9_ thin film on ITO. The thickness of layer-Cs_3_Sb_2_I_9_ was about 170 nm (Supplementary Fig. 13a). The film had RMS roughness of 36.1 nm, and average grain size = 290 ± 15 nm, as calculated using the ASTM intercept procedure (Supplementary Fig. 13b–d)^60^. The XRD of layer-Cs_3_Sb_2_I_9_ had high intensity diffraction peak at 25.8°, which is similar to previously reported spectra of Cs_3_Sb_2_I_9_ with layer structure (Supplementary Fig. 14)^38,39^. These results indicated that the layer-Cs_3_Sb_2_I_9_ film was formed. We fabricated an Au/layer-Cs_3_Sb_2_I_9_/ITO device, which showed bipolar resistive switching behavior (Supplementary Fig. 15). To compare the switching speed of the devices that used different forms of Cs_3_Sb_2_I_9_, we applied voltage pulses (6 V) with different pulse widths. The resistance change (Δ*R*) was defined as the ratio between initial resistance and changed resistance after pulse application. The device that used layer-Cs_3_Sb_2_I_9_ RSM showed little resistance change at pulse width of 100 ns, whereas the device that used dimer-Cs_3_Sb_2_I_9_ showed resistance change at pulse width of 20 ns (Fig. 4d). These observations matched well with our DFT calculation results. In addition, we checked the current changes of layer-Cs_3_Sb_2_I_9_ RSM device by the application of voltage pulses (20 ns) higher than 6 V. When voltage pulses (6, 8, and 10 V) were applied, there was a slight increase of current levels according to voltages, but the change was not enough for memory operation (Supplementary Fig. 16). These results indicated that the dimer-Cs_3_Sb_2_I_9_ could be operated with fast switching speed. A comparison between previous HP RSMs and this device is made (Supplementary Table 2).

In summary, we designed the HPs for Pb-free RSM with fast switching speed by using a combined method of first-principles-based high-throughput screening and experimental verification. A total of 696 compounds with four crystal structures were subjected to a four-step screening process that considered three descriptors (*E*_form_, DFE [*V*_*X*_], and *E*_mig_ [*V*_*X*_]) to identify candidate materials for RSM. The process identified Cs_3_Sb_2_I_9_ with dimer structure as the best compound for Pb-free RSM with fast switching speed. To verify the calculation results, we fabricated RSM device that used the selected dimer structure of Cs_3_Sb_2_I_9._ The fabricated RSM was operated with ultra-fast switching speed (~20 ns) compared to the RSM device that used the layer-Cs_3_Sb_2_I_9_ (>100 ns); these measurements matched well with DFT calculation results. These results demonstrated the feasibility of reliable Pb-free HP memory device with ultra-fast switching speed for RSM. We believed that the combined high-throughput screening and experimental verification could be used to design materials for HP RSM devices.

## Methods

### Materials

Dimethylformamide (DMF, 99.8% purity), cesium iodide (CsI, 99.999% purity), antimony iodide (SbI_3_, 98%, purity), and ITO-coated glass substrates were purchased from Sigma-Aldrich.

### Device fabrication

Precursor solution for dimer-Cs_3_Sb_2_I_9_ was prepared by dissolving CsI (0.75 mmol) and SbI_3_ (0.5 mmol) in DMF (1 ml) solvent overnight at room temperature. Then, the prepared solution was filtered using a polyvinylidene fluoride (PVDF) filter with 0.45-μm pore-size. An ITO-coated glass substrate was sequentially cleaned in acetone, isopropyl alcohol (IPA), and distilled water for 15 min each, then exposed to UV/O_3_ for 20 min. Then Cs_3_Sb_2_I_9_ thin-film was deposited in a glovebox by using the antisolvent dripping method. The precursor was coated at 2000 rpm for 30 s with acceleration time of 5 s. IPA as antisolvent was dropped on the rotating substrate after 2–4 s during the spin-coating process. The deposited film was dried at 100 °C for 10 min to remove any residual elements. Au top electrode with a diameter of 100 μm was deposited on Cs_3_Sb_2_I_9_ film by thermal evaporation. Layer-Cs_3_Sb_2_I_9_ was formed using a mixed solution of CsI (0.75 mmol), SbI_3_ (0.5 mmol), and HCl (30 μl) in DMF^39^. The prepared solution was coated under the same condition as dimer-Cs_3_Sb_2_I_9_, except for the dropping time of antisolvent. The antisolvent was dropped on the rotating substrate after 8–10 s, then the film was annealed at 230 °C for 10 min. The other processes for device fabrication proceeded in the same way as dimer-Cs_3_Sb_2_I_9_.

### Characterization

The morphology and microstructure of Cs_3_Sb_2_I_9_ were measured using a field-emission SEM (JSM 7800, JEOL) and AFM (NX 10, Park systems). The crystal structure of Cs_3_Sb_2_I_9_ was determined using XRD (D/MAX-2500, Rigaku). The surface of Cs_3_Sb_2_I_9_ was investigated using XPS (ESCA LAB250, Thermo Scientific). The electrical characteristics of the fabricated device were measured using a semiconductor parameter analyzer (4200A-SCS, Keithley). The switching speed was measured using a function generator (33600A, Keysight) and an oscilloscope (TDS 5054, Tektronix). Grain size (*G*) was calculated as *G* = 6.643856 log_10_(*P*_*L*_) − 3.288, where *P*_*L*_ is the number of intersections of grain boundary per unit length of the test line^60^. *P*_*L*_ was determined as *P*_*L*_ = *P*_*i*_/(*L*/*M*), where *P*_*i*_ is the number of intercepts of test lines, *L* is the length of test lines, and *M* is the magnification.

### Calculation methodology

We conducted the DFT calculations-based high-throughput screening method to design the optimal HP for RSM. DFT calculations were performed using the projector augmented wave method^63^ with the Perdew–Burke–Ernzerhof functional for exchange and correlation potentials^64,65^, which were implemented in the Vienna Ab-initio Simulation Package (VASP) code^66,67^. To calculate the formation energy of a perovskite structure with combination of compounds, the force convergence criterion in structure optimization was set to be 0.05 eV/Å at the first step and recalculated as 0.01 eV/Å at the second step, with kinetic energy cutoff of 500 eV. The van der Waals correction by using the DFT-D3 method was utilized to consider the interlayer interactions^68^. The calculations of halide vacancy defect-formation energy were performed with the system size, which did not affect the calculation results (8 f.u. for ABX_3_ and A_2_BX_4_, and 2 f.u. for dimer-A_3_B_2_X_9_ and layer-A_3_B_2_X_9_). Gamma centered Monkhorst–Pack *k*-point sampling was used to obtain the relaxed structures (2 × 2 × 2 grids for A_2_BX_4_, ABX_3_, and dimer-A_3_B_2_X_9_; 2 × 2 × 1 grids for layer-A_3_B_2_X_9_)^69^. To calculate the migration energy barrier of halide vacancy defect, the nudged elastic band (NEB) method was utilized in the last step^70^. The NEB method considered a 2 × 2 × 2 supercell for ABX_3_, and 1 × 2 × 1 supercell for both dimer- and layer-A_3_B_2_X_9_.

## Supplementary information

Supplementary Information

## Data Availability

The data that support the findings of this study are available from the corresponding author upon reasonable request.
