# Supplementary material for: Designing zero-dimensional dimer-type all-inorganic perovskites for ultra-fast switching memory
Source: Nat Commun. 2021 Jun 10;12:3527. doi: 10.1038/s41467-021-23871-w (PMC8192534; doi:10.1038/s41467-021-23871-w)
Supplement: Supplementary file 1 — Supplementary Information [file 41467_2021_23871_MOESM1_ESM.pdf]

## Supplementary Information

# Designing zero-dimensional dimer-type all-inorganic perovskites for ultra-fast switching memory

Youngjun Park[1], Seong Hun Kim[1], Donghwa Lee[1,2]*, and Jang-Sik Lee[1]*

[1]Department of Materials Science and Engineering, Pohang University of Science and Technology (POSTECH), Pohang 37673, Korea

[2]Division of Advanced Materials Science, Pohang University of Science and Technology (POSTECH), Pohang 37673, Korea

These authors contributed equally: Youngjun Park and Seong Hun Kim

Corresponding authors' E-mail: jangsik@postech.ac.kr, donghwa96@postech.ac.kr

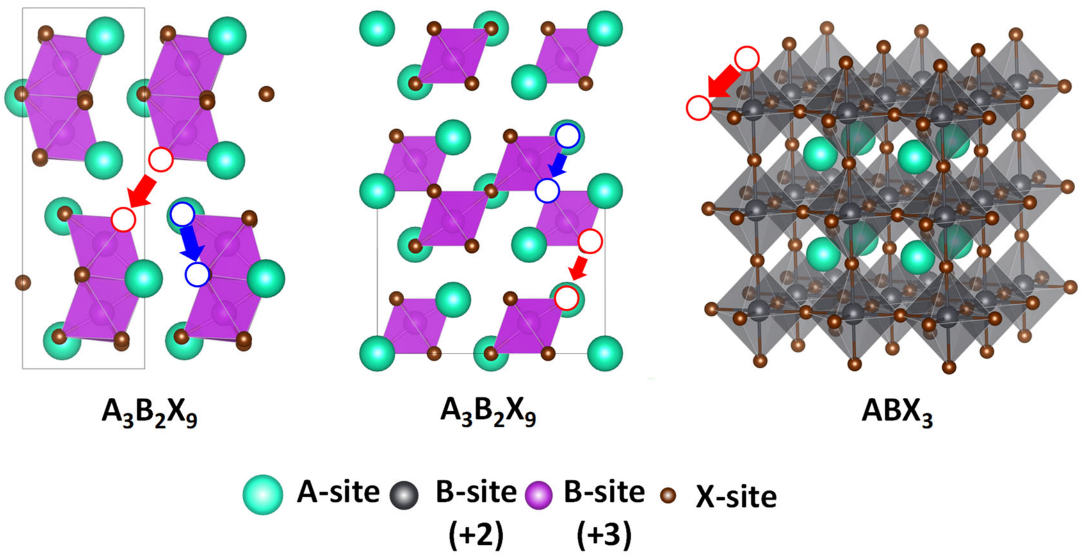


**Supplementary Figure 1.** Vacancy migration paths of HPs. Red circles and arrows represent inter-octahedron migration path. Blue circles and arrows represent intra-migration path. The rectangular boxes in schematic structures are the unit cell of crystal structure.

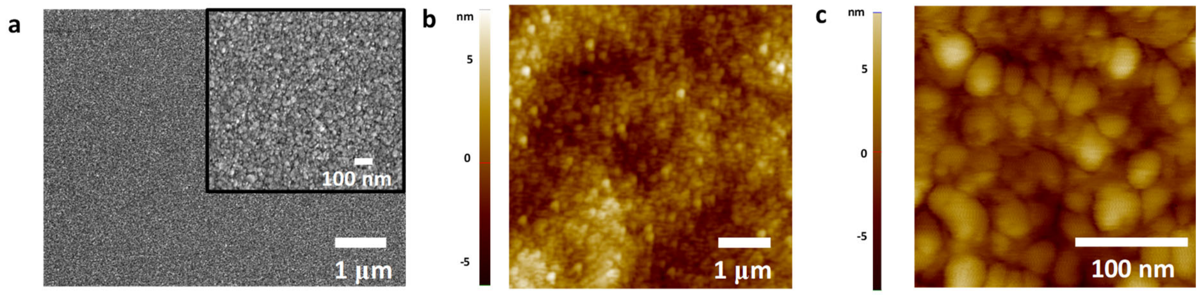


**Supplementary Figure 2.** (a) Plan-view SEM image of the dimer-$Cs_3Sb_2I_9$ thin film (inset: magnified image). (b) AFM image of the surface of the dimer-$Cs_3Sb_2I_9$. (c) Magnified AFM image of dimer-$Cs_3Sb_2I_9$.

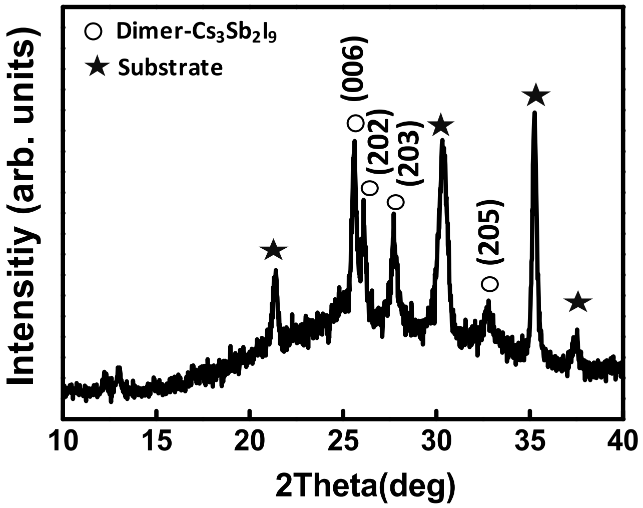


**Supplementary Figure 3.** XRD pattern of the dimer-$Cs_3Sb_2I_9$ thin film on ITO.

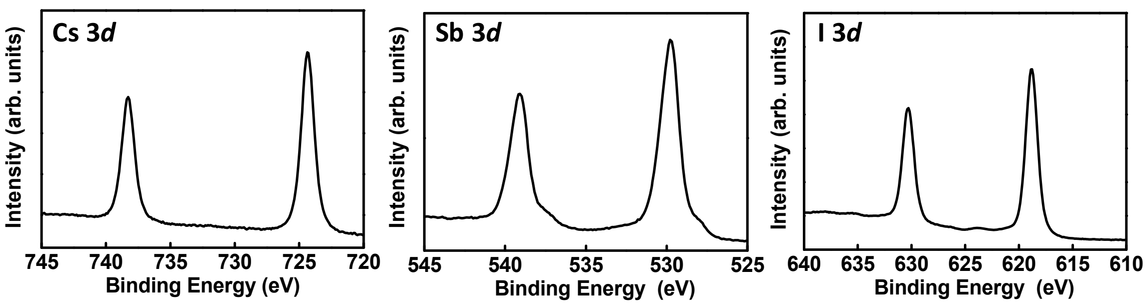


**Supplementary Figure 4.** XPS spectra of dimer-$Cs_3Sb_2I_9$ thin film.

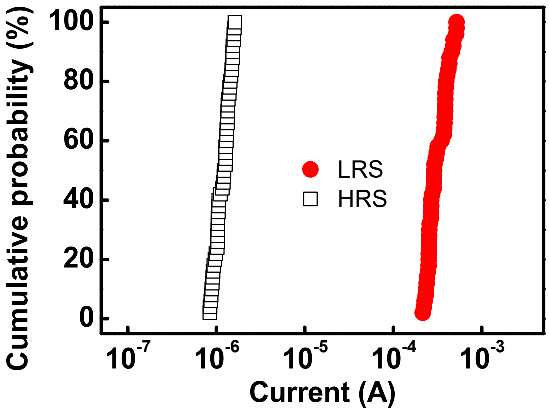


**Supplementary Figure 5.** Cumulative probability distributions of the resistance states during 50 consecutive voltage sweeps (0 V → 1 V → 0 V → -1 V → 0 V). The resistances of LRS and HRS are obtained at a read voltage of 0.1 V.

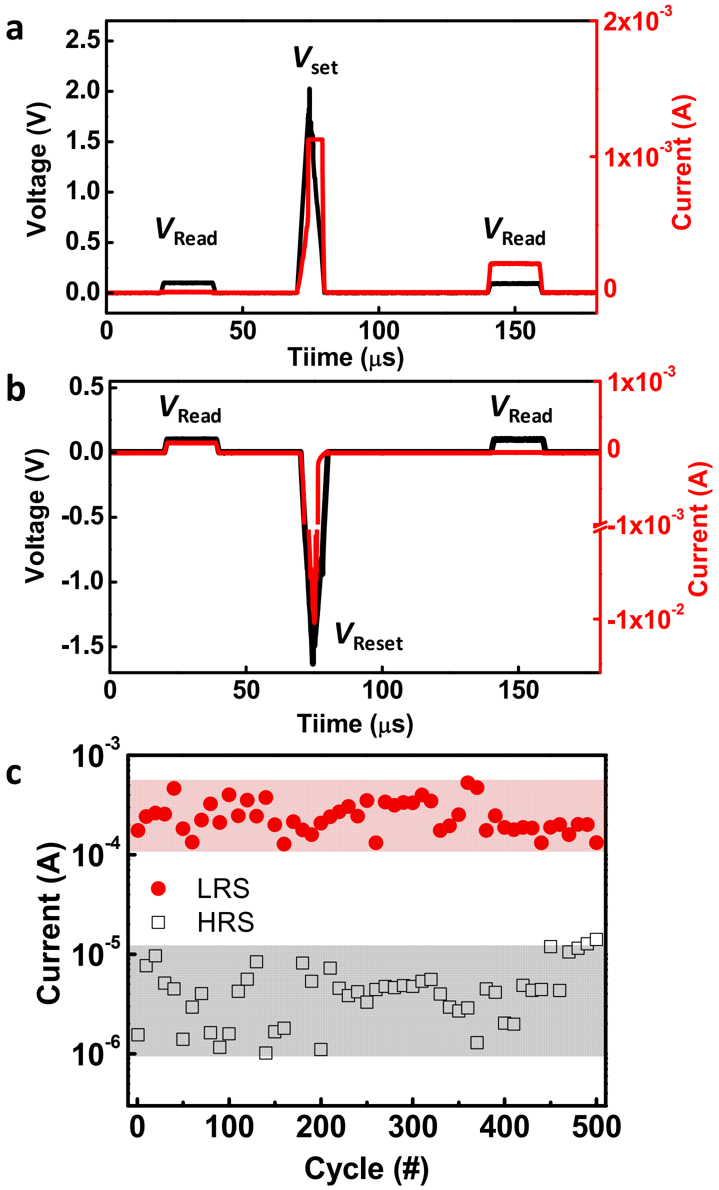


**Supplementary Figure 6.** Application of voltage pulses for (a) set and (b) reset processes. Positive triangular pulses that had a peak voltage of 2 V for the set process, and negative triangular pulses that had a peak of -1.6 V for the reset process, and the change of current level is measured under a read voltage of 0.1 V. (c) Endurance of dimer-$Cs_3Sb_2I_9$ RSM device.

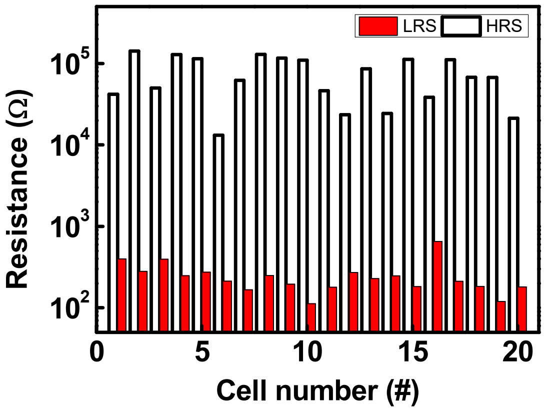


**Supplementary Figure 7.** Device-to-device variation of the dimer-$Cs_3Sb_2I_9$ RSM device. The resistance states of 20 devices are measured at a read voltage of 0.1 V.

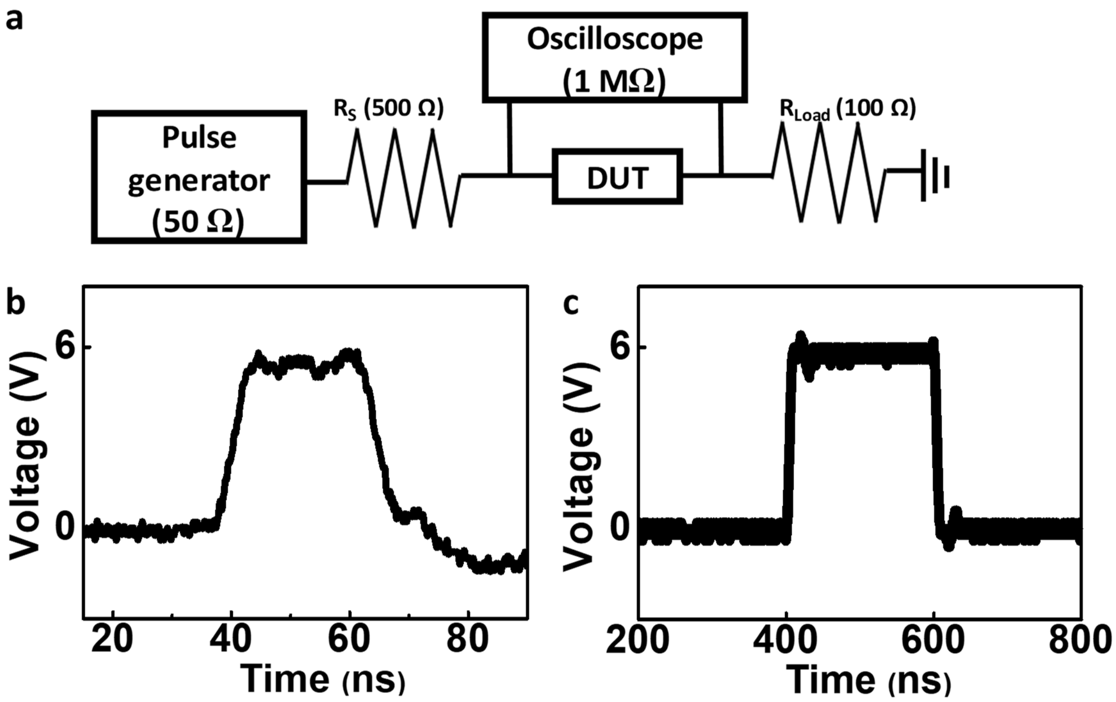


**Supplementary Figure 8. a.** Measurement scheme for pulse application. The waveforms of applied **b.** 20 ns and **c.** 200 ns pulses.

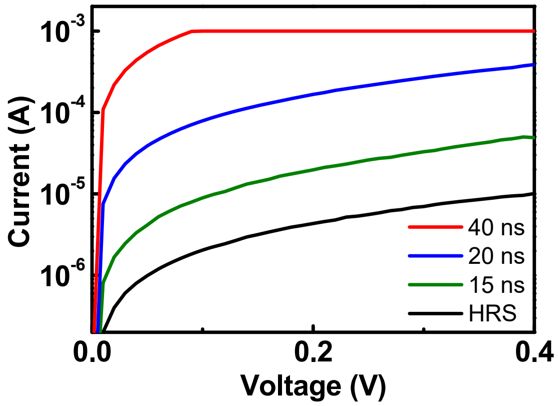


**Supplementary Figure 9.** *I-V* characteristics of the dimer-$Cs_3Sb_2I_9$ RSM by applying set pulses (6 V) with different pulse widths (15 ns, 20 ns, and 40 ns).

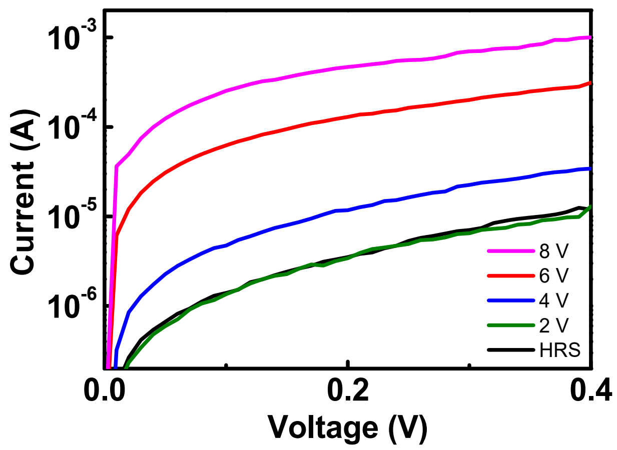


**Supplementary Figure 10.** *I-V* characteristics of the dimer-$Cs_3Sb_2I_9$ RSM by application of set pulses (20 ns) with different voltage amplitudes (2 V, 4 V, 6 V, and 8 V).

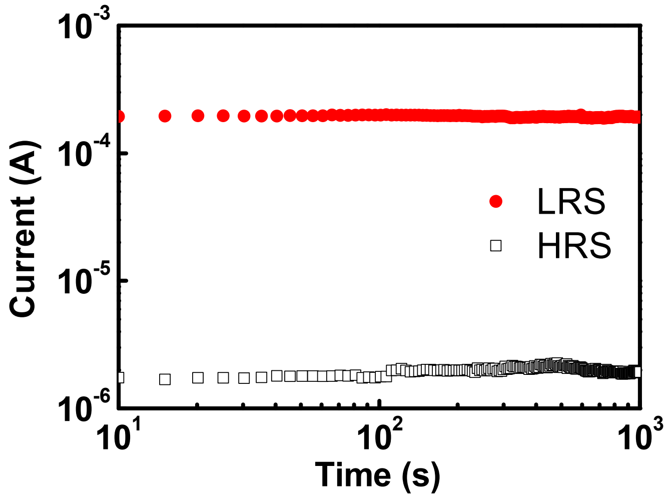


**Supplementary Figure 11.** Data retention characteristics of the dimer-$Cs_3Sb_2I_9$ RSM at LRS and HRS.

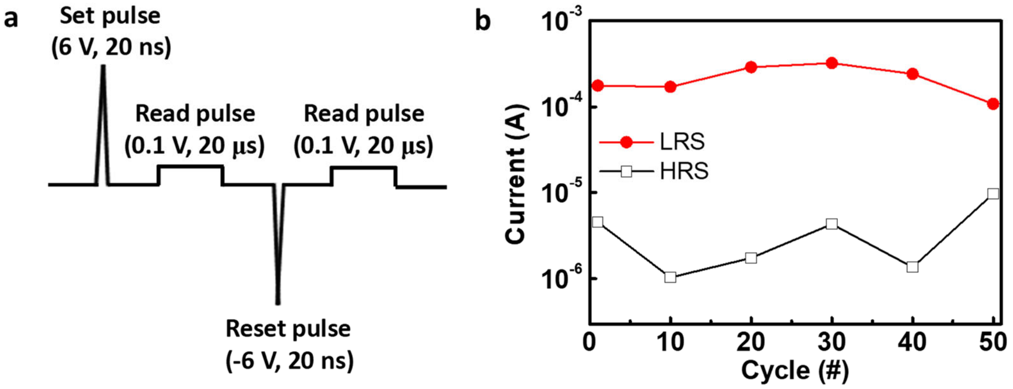


**Supplementary Figure 12.** (a) Applied pulse scheme. (b) Endurance of the dimer-$Cs_3Sb_2I_9$ RSM using 20 ns pulse application.

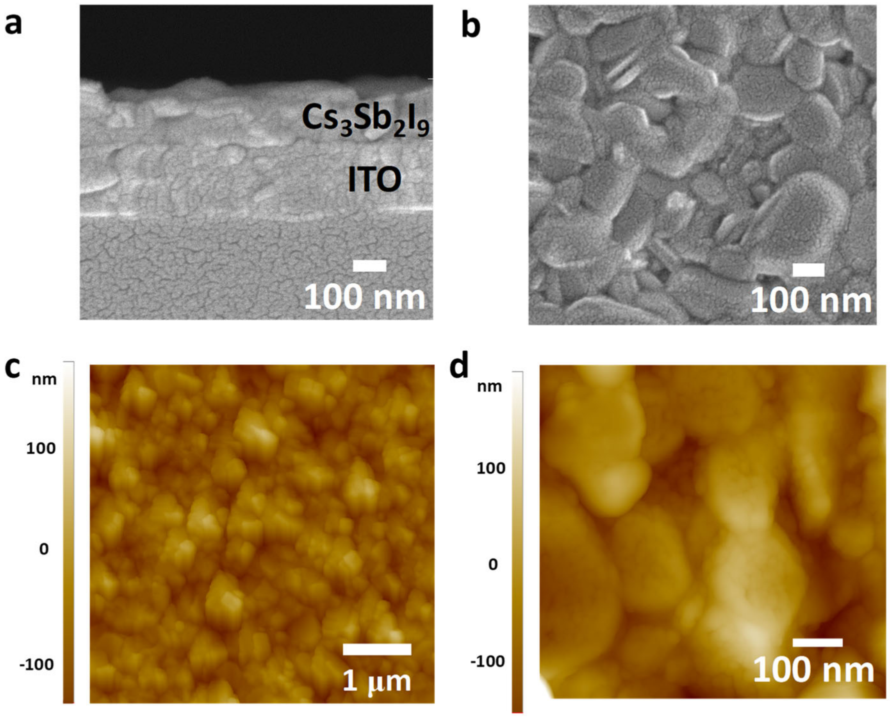


**Supplementary Figure 13.** (a) Cross-sectional SEM image of the layer-$Cs_3Sb_2I_9$ film. (b) Plan-view SEM image of the layer-$Cs_3Sb_2I_9$ film. (c) AFM image of the surface of the layer-$Cs_3Sb_2I_9$ film. (d) Magnified AFM image of layer-$Cs_3Sb_2I_9$.

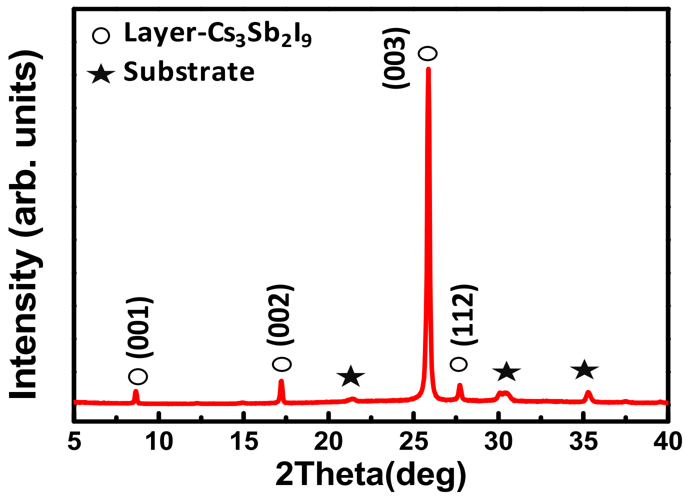


**Supplementary Figure 14.** XRD pattern of layer-$Cs_3Sb_2I_9$ thin film on ITO.

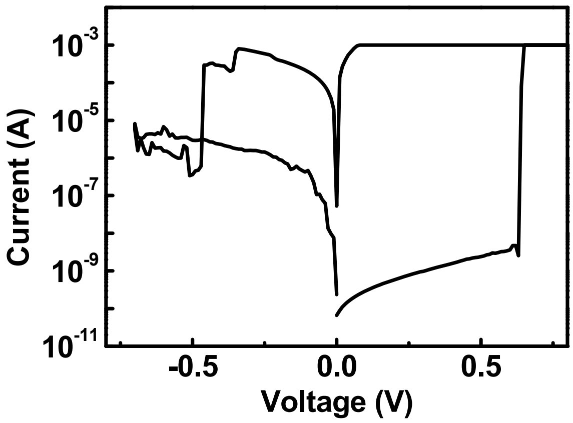


**Supplementary Figure 15.** *I-V* characteristics of layer-$Cs_3Sb_2I_9$ RSM device.

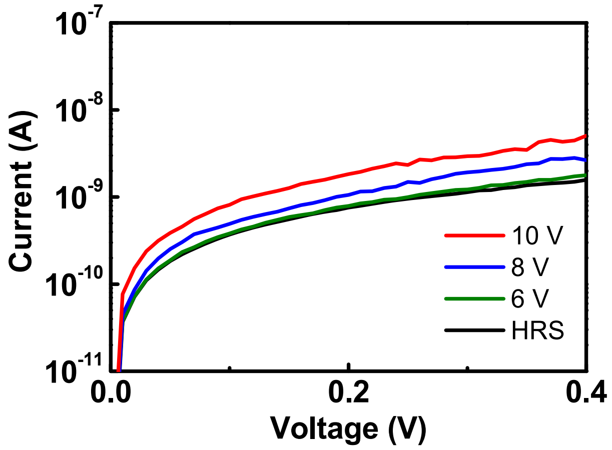


**Supplementary Figure 16.** *I-V* characteristics of the layer-$Cs_3Sb_2I_9$ RSM by application of set pulse (20 ns) with different voltage amplitudes (6 V, 8 V, and 10 V).

**Supplementary Table 1.** Vacancy migration barriers of seven compounds according to two migration paths at final steps.

| Compound name | Inter-octahedron barrier (eV) | Intra-octahedron barrier (eV) |
|---|---|---|
| $Cs_3Sb_2I_9$(layer) | 0.54 | 0.24 |
| $Cs_3Sb_2I_9$(dimer) | 0.47 | 0.37 |
| $Cs_3Bi_2I_9$(layer) | 0.45 | 0.25 |
| $FA_3Sb_2I_9$(dimer) | 1.2 | 0.79 |
| $CsPdBr_3$ | 0.44 | 0.14 |
| $GA_3Sb_2Br_9$(layer) | 0.96 | 0.34 |
| $FA_3Sb_2I_9$(layer) | 1.04 | 0.32 |

**Supplementary Table 2.** Switching speed of HP RSM devices.

| Device structure | Switching speed | Reference |
|---|---|---|
| **Au/$Cs_3Sb_2I_9$/ITO** | 20 ns | This work |
| **Al/$CH_3NH_3PbI_3$:PVAm·HI/ITO** | 50 ns | 1 |
| **Au/$CH_3NH_3PbI_3$/FTO** | 60 ns | 2 |
| **Au/$(CH_3NH_3)_3Bi_2I_9$/ITO** | 100 ns | 3 |
| **Pt/Zr-doped $CH_3NH_3PbI_3$/FTO** | 480 ns | 4 |

1. Cao X., *et al.* Enhanced switching ratio and long-term stability of flexible RRAM by anchoring polyvinylammonium on perovskite grains. *ACS Appl. Mater. Interfaces* **11**, 35914-35923 (2019).

2. Ma H., *et al.* Interface state-induced negative differential resistance observed in hybrid perovskite resistive switching memory. *ACS Appl. Mater. Interfaces* **10**, 21755-21763 (2018).

3. Hwang B. & Lee J.-S. Lead-free, air-stable hybrid organic–inorganic perovskite resistive switching memory with ultrafast switching and multilevel data storage. *Nanoscale* **10**, 8578-8584 (2018).

4. He Y., *et al.* Impact of chemical doping on resistive switching behavior in zirconium-doped $CH_3NH_3PbI_3$ based RRAM. *Org. Electron.* **68**, 230-235 (2019).
